# Distortion product otoacoustic emissions in sheep before and after hyperinsulinemia induction

**DOI:** 10.1016/S1808-8694(15)31086-7

**Published:** 2015-10-19

**Authors:** Francisco Carlos Zuma e Maia, Luiz Lavinsky, Roseli Oliveira Möllerke, Marcos Eugenio Soares Duarte, Daniela Peres Pereira, Juliana Elert Maia

**Affiliations:** 1 M. Sc. PhD student - Graduate Program in Surgery - Otolaryngology - Universidade Federal do Rio Grande do Sul; 2 PhD in Otolaryngology, Associate Professor Universidade Federal do Rio Grande do Sul - Medical School; 3 PhD. Veterinary Professor - Veterinary School - Universidade Federal do Rio Grande do Sul; 4 Veterinary Medicine Student - Research Center - Universidade Federal do Rio Grande do Sul; 5 Speech and Hearing specialist; 6 Nutrition Student - Universidade Federal do Rio Grande do Sul

**Keywords:** otoacoustic emissions, hyperinsulinemia, distortion product

## Abstract

Transient evoked otoacoustic emissions and distortion product otoacoustic emissions have gained significant importance in the identification of cochlear alterations.

**Aim:**

To record distortion product thresholds through the monitoring of otoacoustic emissions in normal conditions and in the presence of electrophysiologic changes in cochlear outer hair cells in sheep after hyperinsulinemia induction.

**Material and methods:**

Experimental study, with seven sheep in the control group and seven in the study group. Insulin and glucose concentrations were measured simultaneously for the recording of distortion product otoacoustic emission every 10 minutes, all the way to 90 minutes. The control group received saline solution, and the study group received a bolus injection of 0.1 U/kg of regular human insulin.

**Results:**

There was a significant reduction in distortion product thresholds in the study group when compared to the control group at frequencies greater than 1,500Hz and after 60 minutes (P < 0.001).

**Conclusion:**

This study established distortion product otoacoustic emission thresholds in sheep with constant reproducibility, demonstrating that the method is adequate for use in audiology and otology investigations. Results also fully confirm that acute hyperinsulinemia may cause important changes in these thresholds.

## INTRODUCTION

In the past decade, evoked otoacoustic emissions (EOE) were used to study the mechanical aspects of the cochlear function. Between the two basic EOE - Evoked Transient (ETEOE) and those by distortion product (DPEOE) - the former are the ones most employed in clinical practice, capable of showing initial cochlear alterations, which are not detected in tonal audiometry nor in other conventional tests, in Ménière's disease and in other common inner ear disorders.

There are evidences of cochlear and vestibular involvement, even in the very first stages of metabolic alterations associated with glucose and insulin^1^, ^2^, ^3^, ^4^, ^5^. Research with animal models focused on endocochlear disorders has revealed that the inner ear practically does not store energy; it depends on the oxygen supply and glucose perfusion to keep its intense level of activity. Therefore, changes in metabolism or in blood flow have a great likelihood of changing inner ear homeostasis^6^, and it is likely that studying these changes we can shed some light on the relationship between glucose metabolism disorders and inner ear problems.

The use of sheep for experimental purposes was initially proposed by Lavinsky & Goycoolea^7^ for otology research, based on our own prior work^8^^,^^9^ which revealed significant similarities between sheep and humans in terms of ear anatomy, histology and morphometry, especially regarding the size of the structures. Because of this very similarity, sheep are especially used for surgical studies and otologic neurophysiology studies.

Starting from the operational hypothesis that we can use EOE in otologic investigations in sheep, the goal of the present study was to record distortion product thresholds, as well as the electrophysiological modifications on cochlear outer hair cells (OHC) after we cause a metabolic change to the cochlea, establishing acute hyperinsulinemia.

## MATERIALS AND METHODS

The present experimental study followed the international principles of animal handling^10^ and was approved by the Ethics Committee of the Graduate Program of our Institution (Protocol n°. 04-051).

The size sample was based on a pilot study that included three animals in the study group and three in the control group. The animals that reached indices above 50% of reproducibility on transient EOE and with distortion product (DP) at the time zero of our experiment were included in the study.

Fourteen male Textel sheep, with average weight of 40kg and age around 18 months, were divided and randomized in two groups (control and study); the experiments did not cause any discomfort to the animals, since they were under sedation and general anesthesia. Since this has been an acute observational study, the animals were not slaughtered, and returned to their place of origin at the end of the experiment.

### Induction of acute hyperinsulinemia and anesthesia

In the Control Group (n = 7), after 48 hours of fasting, each animal received 20ml of endovenous saline solution. The EOE was recorded in time zero and at every 10 minutes thereafter, up to a total of 90 minutes, concurrently with blood withdrawing for insulin and glucose measuring. In the Study Group (n = 7), after a 48 hour fasting, the animals received an intravenous bolus injection of insulin (0.1 U/kg diluted in 10ml of saline solution). EOE was also recorded at time zero and at every 10 minutes thereafter, up to a total of 90 minutes, concurrently with blood withdrawing for insulin and glucose measurements.

The animals were sedated using intramuscular acepromazine (Univet, São Paulo, SP) 500 mg/kg. For anesthesia induction we used 15 mg/kg of intravenous thiopental (Abbott, Rio de Janeiro, RJ). To maintain anesthesia we used 600 mg/h of sodium thiopental in a continuous infusion pump (Nutrimat II, B. Braun, São Gonçalo, RJ). We used local anesthesia in the ear, close to the pinna, with 75 g of 0.75% of bupivacaine chloridrate (Astra, Cotia, SP).

At 10 minutes after pre-medication the animal was induced with thiopental, intubated with a 8–9mm tracheal tube and connected to the anesthesia cart in a closed loop circuit (Modulus 4000, Narcosul, Porto Alegre, RS), receiving 100% oxygen. The following anesthesia parameters were monitored: oxygen saturation, heart rate (Biox 3700e, Ohmeda, Louisville, CO, EUA), current volume, respiratory rate (Ventcare, São Paulo, SP) and rectal temperature (Sontemp 400/700, Sheridan, WY, EUA).

### Surgery to expose the external acoustic meatus

**Figure 1:**
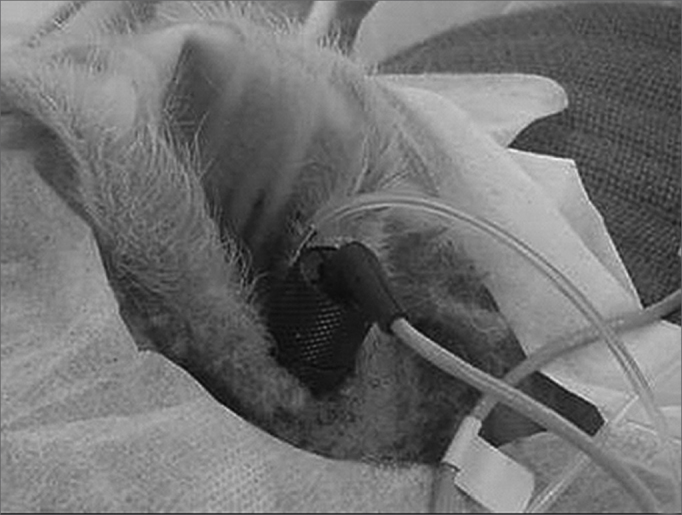
Stable probe fitting and fixing to the external auditory meatus.

We used an electrical scalpel (BMO-870, Medcir, São Paulo, SP), for the incision after local anesthesia with 0.75% bupivacaine chloridrate at about 2cm pre-auricular, intending to enhance visualization of the external acoustic meatus and tympanic membrane, and to place EOE probe (Figure 1).

### EOE recording methods

During the recordings, the body temperature of each animal was kept at 37° 0.5°C, using a thermal mattress (Termway). Room noise levels during the procedure were never above 65.5 dB (A).

**Figure 2:**
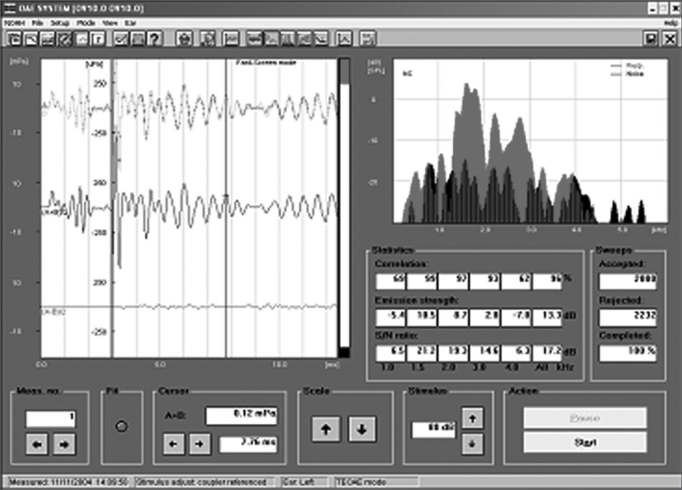
Evoked transient otoacoustic emissions recording in sheep - time zero.

ETEOE response (Figure 2) was recorded by a Capella model Madsen device, coupled to a Toshiba Satellite notebook computer. The parameters used were carried out in the fast screen mode, with a 40 microseconds duration click at 80 dB SPL, with condensation polarity. For data analysis we used only the segments of records on the window from 3 to 12.5ms.

**Figure 3:**
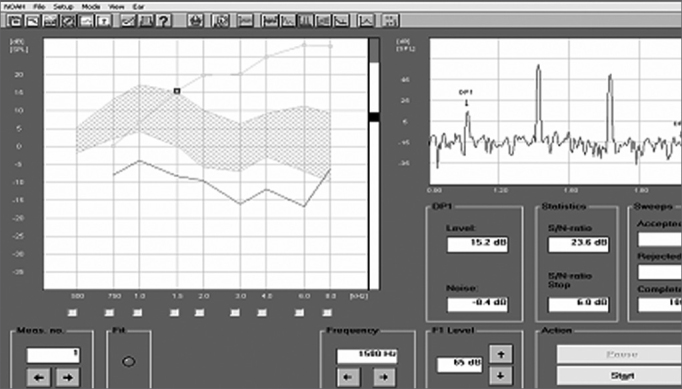
Distortion product otoacoustic emissions recording in sheep - time zero.

DPEOE (Figure 3) were recorded by the same equipment used for ETEOE. The parameters used were PD1 = 2F1 - F2 (F = sound stimulus frequency), with F1: F2 = 1.22. Intensity levels were: L1 = 65 and L2 = 55 Db (where L = sound stimulus intensity). For DPEOE analysis we used the intensity thresholds (dB) in the following frequencies: 750; 1,000; 1,500; 2,000; 3,000; 4,000; 6,000 and 8,000 Hz.

### Blood glucose and insulin measurementsin

Each animal was submitted to jugular vein punch for blood harvesting and later glucose analysis by the glucoseoxidase enzymatic colorimetric method, using the Labtest kit and insulin by immunoassay and electrochemoluminescence, in the Modular Analytics E170 (Roche) analyzer, at 10 minute intervals, up to a total of 90 minutes.

### Statistical analysis

Data were transferred to a computerized statistical program - Statistical Package for the Social Sciences (SPSS), version 13.0 - for analysis. For the pilot group, we used the t Student test to compare the two groups with magnitude of two standard deviations effect, α = 0.05 and 90% power, with an estimate of six animals per group.

During the experiment, we carried out the binomial test to assess the occurence of minimal values in DP thresholds, in the frequencies of 750, 1,000; 1,500; 2,000; 3,000; 4,000; 6,000 and 8,000 Hz, at the times of 60, 70, 80 and 90 minutes, the same thing happened between the control and study groups.

## RESULTS

We started the study with 18 animals. Throughout the experiment development, two animals were taken off the study on account of anatomical reasons associated with the external acoustic meatus, one animal because of unfavorable anesthetic conditions which led to animal restlessness during the experiment resulting in EOE probe shifting and one death during anesthesia.

All the animals were very well behaved during all the pre-operative procedures (anesthetic induction). There were no cases of hemorrhage, only one case of death by pulmonary aspiration in the control group. Total anesthetic recovery happened in about 2 hours, without accidents. Following that, the animals were transported to their place of origin, where they had their operative wound taken care of.

The method employed did cause the hypoglycemia and hyperinsulinemia we aimed at (Figure 4A and Figure 4B).

**Figure 4a:**
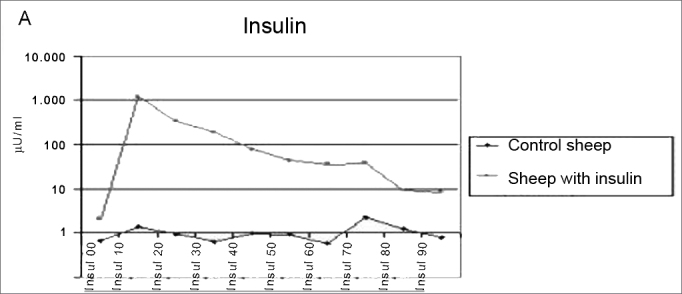
Insulin levels mean value in the control and study groups during 90 minutes.

**Figure 4b:**
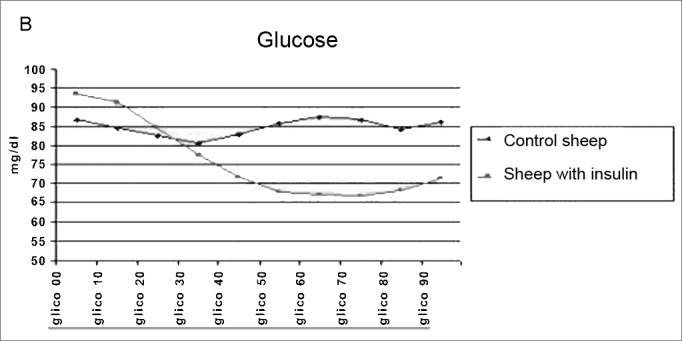
Glucose levels mean value in the control group during 90 minutes.

**Figure 5:**
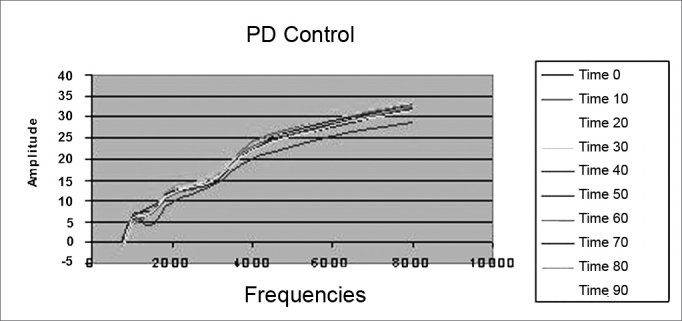
Distortion product otoacoustic emissions mean level in the control group during 90 minutes. PD = distortion product.

There were no changes in DP thresholds in the control group in relation to glucose and insulin levels during 90 minutes (Figure 5).

**Figure 6:**
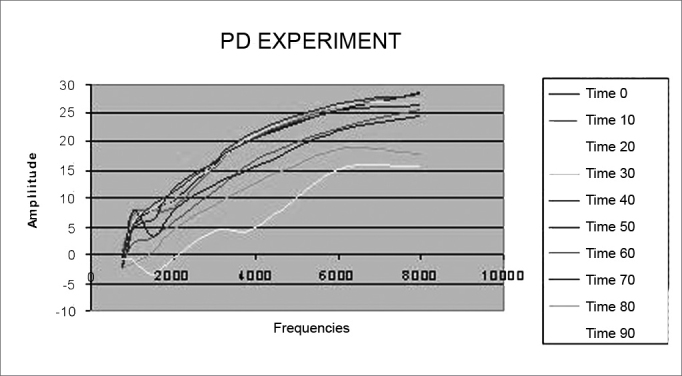
Distortion product otoacoustic emission levels mean value in the study group during 90 minutes. PD = distortion product.

On Figure 6, there is a reduction in DP thresholds in the frequencies from 750 to 8,000 Hz in the study group, according to time.

Table 1 shows the minimum thresholds observed for DP in four time points, starting at 60 minutes of recording after insulin injection in the study-group for the eight frequencies studied (Hz).

**Table 1:** Minimum values for distortion products in four recording periods (min) after insulin injection in the study group, listing the eight frequencies (Hz) investigated^a^.

Frequency (Hz)	Control					Study		
	60 min	70 min	80 min	90 min	60 min	70 min	80 min	90 min
750	−6.2	−16.5	−19.8	−19.1	−8.2	−6.3	−16.9	−20.0
1.000	−1.6	−4.4	−2.3	0.5	−23.1	−0.1	−9.3	−19.5
1.500	−4.6	−3.7	−7.4	−4.0	−26.6	−21.6	−17.2	−30.2
2.000	−5.5	−0.5	2.2	−1.8	−32.9	−21.9	−22.9	−31.5
3.000	5.2	3.8	5.9	6.1	−25.1	−17.8	−18.7	−34.3
4.000	10.6	8.1	8.4	7.8	−22.7	−27.7	−24.3	−50.5
6.000	23	19.5	21.4	22	−11.8	−14.6	−8.6	−18.3
8.000	18.9	18.4	23.8	25.8	−6.3	−12.9	−19.9	−25.5

a Binomial test for the homogeneous occurence of minimum values between the control (n = 7) and study (n = 7) groups (P < 0.001).

## DISCUSSION

There are a number of techniques that may be used to monitor auditory function. TEEOE and DPEOE, as the representatives of OHC's working conditions, are recent clinical tests and are taking on significant importance in the identification of cochlear alterations, especially because it is an objective, non-invasive test, which can be easily employed^11^^,^^12^. Even not establishing an auditory threshold and not replacing tonal audiometry, immitanciometry or BERA, these new tests may detect initial signs of cochlear damage^13^.

In the sample used in the control group of the present investigation, we obtained DPEOE from all the animals. This finding confirms the fact - already broadly shown in many a research, e.g. Robles et al.^14^ in chinchillas and those from Schrott^15^ in mice - that DPEOE are present in normal ears, therefore, the Corti organ's hair cells, when healthy generate DP. Also in humans, Lopes Filho^16^ and Coube^17^ obtained DPEOE responses in 100% of the ears tested.

We must stress that, during the 10 recordings carried out in each animal of the control-group, we observed a constant reproducibility in the findings, showing that the results are reliable and, moreover, they may help establish a standard concept of normality for this study in bovines.

Animals currently used for research and training, such as dogs, cats and monkeys, have a different size when compared to man, and are difficult to handle in captivity, because they are aggressive and susceptible to diseases. Moreover, often times, these animals are expensive and not easily available; many are pets, and their use in training and research may bring about psychosocial repercussions and cause conflicts with animal protection entities^7^. For its robustness, availability, ease handling, resistance and low cost, sheep are animals which are especially suitable for training and experiments in otology. Other papers^7^, ^8^, ^9^ show significant similarities with human ears, such as in anatomy, hystology and morphology, most especially as far as the size of the structures are concerned, which really facilitates the study of surgical order, as well as those associated with otologic neurophysiology studies. The characteristics and advantages of this type of animal can be equally seen in our study. The same device used in humans can be used in sheep without the need for fittings, when compared to experiments that use smaller animals.

The anesthesia used allowed us to proceed with the experiment and guaranteed a low animal morbi-mortality. Only one animal (from a total of 18) died, because of complications associated with pulmonary aspiration.

In the present investigation, the exposure of the external acoustic meatus through a pre-auricular incision was of fundamental importance in order to let us visualize the tympanic membrane and for the perfect fitting and sealing of the external acoustic meatus, through a proper probe fixation during the EOE test, thus guaranteeing cochlear monitoring (10 recordings from each animal), without any interference during the experiment, in the frequencies of 750; 1,000; 1,500; 2,000; 3,000; 4,000; 6,000 and 8,000Hz, bringing about uniform recording quality and, in consequence, homogeneity in results. Some studies in rats^18^ were unable to properly fix the probe, not allowing for DPEOE recording below 4,000 Hz.

Although it is an objective test, DPEOE amplitude has shown great variability for different individuals, probably because of the different parameters used by each investigator, such as: primary stimuli intensity, difference or not among them and ratio between primary frequencies, and also individual characteristics from each ear, possible central nervous system influence and the presence of other types of EOE.

There are controversies as to the ideal intensities to be employed in F1 and F2. Hauser & Probst^19^ suggest that fixing L2 below L1 must result in an improvement in the signal-to-noise ratio and thus increase small amplitude response detectability in human ears. Animal studies show that the primary levels are more effective when L1 is 5–10 dB higher than L2. Thus, by using stimulation parameters from PD1 = 2F1 - F2, with an F1: F2 = 1.22 ratio, intensity levels from L1 = 65 and L2 = 55 dB and standardization of the Madsen device, Capella model, we were able to record, for the first time, the profile of DPEOE thresholds (DP-gram) in sheep (control group).

By using TEEOE, where stimulation is distributed and acts on a large portion of the basilar membrane, stimulating it entirely and, therefore, having a frequency response spectrum under the influence of the whole cochlea, we used the reproducibility parameter above 50% as inclusion criteria in the time zero study of the experiment in each animal, because from this level on, the findings are not artifacts and, thus, become reliable.

The amplitude of responses captured by DPEOE had a certain growth trend towards high frequencies. Comparing it with studies in humans^17^ and in animal models^18^^,^^19^, they also showed in normal ears a DPEOE function configuration more or less similar to the one hereby reported. It is possible that cochlear tonotopy distribution facilitates this larger amplitude in the high tones, since they are located on the base of the cochlea, a region that is near the EOE capture site. Gorga et al.^20^ suggest some factors as being responsible for the low responses in low frequency stimuli. One of them would be the low signal-to-noise ratio for the low frequencies; another one would be the energy transfer mode by the middle ear system, which has less amplification for low frequency sounds. Also, according to the author, this middle ear characteristic interferes in the low tones transmission, both in the direction from the middle ear to the cochlear (stimulus) as in the inverse direction, from the cochlea to the middle ear (response). These factors seems to bring lower amplitude to the DPEOE captured in the external acoustic meatus, making it very difficult to distinguish between emissions and background noise.

Many authors were able to reduce DPEOE in lab animals, some causing experimental endolymphatic hydrops by occluding the duct and the endolymphatic sac^21^, others^22^ using repetitive microinjections of artificial endolymph.

In our study, we used hyperinsulinemia to electrophysiologically modify cochlear OHC. In order to show the genesis of cochlear damage during this condition of acute hyperinsulinemia, we used a bolus injection of 0.1 U/kg of regular insulin, like the one used in the insulin tolerance test 23, when we assess the glucose reduction test for 15 minutes after insulin injection. Our study helped us obtain a proper response to cause the desired hypoglycemia and hyperinsulinemia. The average values obtained for the analysis of the serum concentrations of insulin and glucose, in our control group, were the same as those found in the veterinary literature^24^^,^^25^ using the commercial kit employed in humans.

The present investigation makes us consider the following aspects as relevant:

– There were no variations in DP thresholds in the control group as far as insulin and glucose levels were concerned in the 10 recordings from each animal and during the 90 minutes of experiment duration, keeping the DP-Gram characteristics stable, which becomes our standard of normality in sheep.
– In the study group, there was a significant reduction in DP thresholds in relation to the control group, and such reduction was much clearer in those frequencies above 1,500 Hz and after 60 minutes (P < 0.001). This information helps us infer that there were electrophysiological alterations on the OHC, especially in the cochlear basal turn.

The OHC account for sound amplification of a specific frequency, in a process called electromobility, resulting from changes in OHC membrane fluids. Therefore, it is possible that the findings from this study result from the acute and elevated action of insulin and that after 60 minutes it starts to impact high frequencies electromobility and also on endolymphatic space ionic patterns.

Although the correlations between cochlear physiological alterations and carbohydrate metabolic disorders are difficult to establish, the DPEOE may eventually become the means for this end. Future studies are necessary in order to clarify the present observation of a significant electrophysiological change in high frequency DP thresholds after 60 minutes of induced acute hyperinsulinemia.

## CONCLUSION

Summarizing, otoacoustic emissions in the study animals had, in the control group, an uniform reproducibility of distortion product thresholds in all the animals, with a curve pattern (ascending) that is similar to what is found in humans. Thus, the method proved to be adequate to be used in otologic and audiologic investigations. Our study also established that acute hyperinsulinemia was capable of causing relevant changes in these thresholds (P < 0.001).
